# COVID-19 vaccine uptake among young adults: Influence of asthma and sociodemographic factors

**DOI:** 10.1016/j.jacig.2024.100231

**Published:** 2024-02-20

**Authors:** Maria Ödling, Niklas Andersson, Sandra Ekström, Niclas Roxhed, Jochen M. Schwenk, Sophia Björkander, Anna Bergström, Erik Melén, Inger Kull

**Affiliations:** a Department of Clinical Science and Education, Södersjukhuset, Karolinska Institutet, Stockholm, Sweden; b Institute of Environmental Medicine, Karolinska Institutet, Stockholm, Sweden; c Centre for Occupational and Environmental Medicine, Region Stockholm, Stockholm, Sweden; d Division of Micro and Nanosystems, KTH Royal Institute of Technology, Stockholm, Sweden; e MedTechLabs, Bioclinicum, Karolinska University Hospital, Solna, Sweden; f Science for Life Laboratory, Department of Protein Science, KTH Royal Institute Technology, Solna, Sweden; g Sachs’ Children and Youth Hospital, Stockholm, Sweden

**Keywords:** Asthma control, allergic disease, birth cohort, comorbidity, population based, SARS-CoV-2, severity, vaccination

## Abstract

**Background:**

Asthma was initially described as a risk factor for severe coronavirus disease 2019 (COVID-19), but the uptake of COVID-19 vaccine among young adults with asthma is not well studied.

**Objective:**

The aims were to assess COVID-19 vaccine uptake among young adults in general and to explore potential determinants including sociodemographic factors and asthma.

**Methods:**

Participants from the population-based birth cohort BAMSE (Barn/Child, Allergy, Milieu, Stockholm, Epidemiology) were included: 4,064 in the study population, 3,064 in a follow-up at age 24 years, and 2,049 in a COVID-19 follow-up (mean age, 26.5 years). Asthma and asthma-associated characteristics were assessed through questionnaires and clinical data. Data on all COVID-19 vaccines registered between January 1, 2021, and February 15, 2023, were extracted from the National Vaccination Register.

**Results:**

In the study population (n = 4,064), 53.9% had ≥3 COVID-19 vaccine doses registered. In the 24-year follow-up population (n = 3,064), vaccine uptake differed in relation to education (*P* < .001). Among the participants with university/college education, 65.7% had an uptake of ≥3 doses of vaccine, compared to 54.1% among the participants with elementary school/high school education. Participants with asthma had decreased odds of receiving ≥3 doses (adjusted odds ratio = 0.62; 95% confidence interval, 0.41-0.92) and ≥2 compared to peers without asthma. Those with uncontrolled disease also had decreased odds of receiving ≥3 doses (adjusted odds ratio = 0.30; 95% confidence interval, 0.13-0.66) and ≥2 compared to participants with controlled asthma.

**Conclusions:**

COVID-19 vaccine uptake among young adults is lower in individuals from households with lower socioeconomic status and among those with asthma, including uncontrolled asthma.

Widespread coronavirus disease 2019 (COVID-19) vaccination has been crucial to protect population health.^1^ When the efficacy and safety of COVID-19 vaccines against severe acute respiratory syndrome coronavirus 2 (SARS-CoV-2) were assessed, it was concluded that most vaccines reduced, or likely reduced, the proportion of individuals with confirmed symptomatic COVID-19. There is strong evidence that some vaccines reduce severe or critical disease compared to placebo.^2^ Though it has been concluded that COVID-19 vaccines offered protection against disease and were key to help stop the pandemic and new virus variants emerging,^3^^,^^4^ willingness to be vaccinated varies. Factors that have been shown to influence vaccination intention and uptake in the adult population include sociodemographic characteristics, age, sex, attitudes toward vaccination, perceptions of risk and susceptibility to COVID-19, and chronic conditions.^5^^,^^6^ In the information from the Public Health Agency of Sweden provided to priority groups regarding COVID-19, risk factors other than age are listed. This information stated that difficult-to-treat and uncontrolled asthma slightly increases the risk of severe COVID-19 symptoms and disease.^7^ How this influences COVID-19 vaccine uptake in those with asthma and asthma-associated characteristics is not well studied, particularly not in a young general population. Our hypothesis was that young adults with asthma were more likely to be vaccinated than peers without asthma.

Age seems to be a factor influencing vaccination intention and uptake, with younger adults playing an important role in virus transmission as a result of the high rates of asymptomatic infections and active social lives.^8^, ^9^, ^10^ This age group has been identified by public health authorities as a key target for vaccine uptake.^9^ Therefore, investigating factors that influence young adults to be vaccinated against COVID-19 is a high-priority task. The aims of the present study were thus to assess COVID-19 vaccine uptake among young adults in general and to explore potential determinants including sociodemographic factors and asthma.

## Methods

### Study design and study population

**Fig 1:**
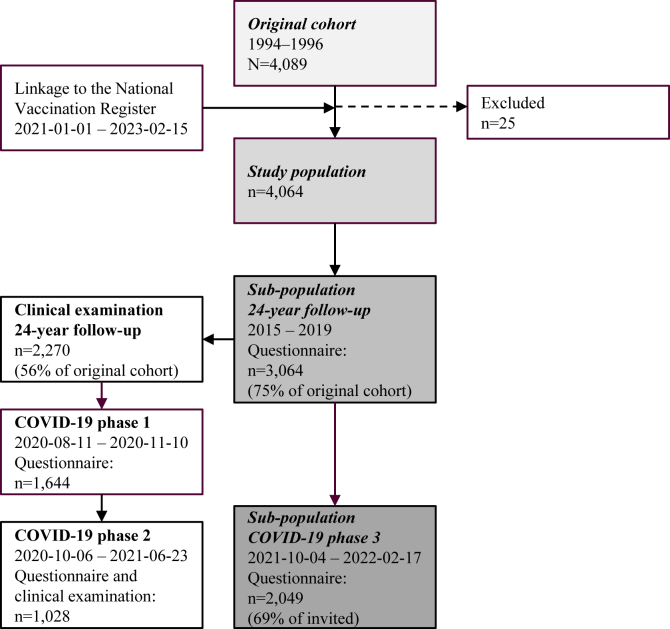
Study flowchart of original cohort (N = 4,089), study population (n = 4,064), subpopulation 24-year follow-up (n = 3,064), and subpopulation COVID-19 phase 3 (n = 2,049).

Data were gathered from the ongoing population-based birth cohort BAMSE (Barn/Child, Allergy, Milieu, Stockholm, Epidemiology).^11^ The cohort originally included 4,089 participants, who have been followed since birth with repeated questionnaires and clinical examinations, with the primary aim to study risk factors and consequences of allergic diseases (Fig 1). At child age 2 months, a baseline questionnaire was filled out, where information on early life factors was obtained. This was investigated in relation to COVID-19 vaccine uptake in the present study population (n = 4,064, 25 excluded from the original cohort). The latest follow-up was finished in 2019 and is referred to as the 24-year follow-up. The subpopulation 24-year follow-up consisted of individuals who answered the questionnaire (n = 3,064, 75% of the original cohort) and underwent a clinical examination (n = 2,270, 56% of the original cohort) in this follow-up. This was used to study the influence of asthma and lifestyle factors. In 2020, new follow-ups of the BAMSE cohort were initiated to investigate risk factors for COVID-19 and long-term health consequences among young adults. Follow-ups, divided into 3 phases during 2020-22, included 3 web questionnaires and 1 clinical examination. Phases 1 and 2 are presented in detail in Fig 1 and elsewhere.^12^^,^^13^ Phase 3 ran from October 4, 2021, through February 17, 2022 (covering the whole pandemic up to the date of the questionnaire), and an invitation was sent to all participants who had answered the questionnaire at the 24-year follow-up and had provided their email address (n = 2,981).^14^ Of the 2,981 invited participants, 2,049 (69% of the invited) answered the questionnaire and were included in the subpopulation COVID-19 phase 3 to study COVID-19–related factors.

The BAMSE study was approved by the Swedish Ethical Review Authority (approval 93:189, 2016/1380-31/2, 2016/2475-32, 2020-02922, and 2022-00293-02), and all participants provided informed consent.

### Data source of outcome assessment

Information on vaccination against COVID-19 was obtained by linkage to the National Vaccination Register using personal identity numbers.^15^ In Sweden, the first COVID-19 doses were administrated on December 27, 2020, to target groups. We received data on all participants in the study population (n = 4,065) for January 1, 2021, to February 15, 2023 (Fig 1). The Public Health Agency of Sweden is responsible for the vaccination register, which is mandatory; this means that all vaccinations against COVID-19 are recorded.^16^ Using personal identity numbers, it is possible to follow all participants over time.^15^

For most COVID-19 vaccines, a primary series consists of 2 doses.^17^ A first booster dose is an additional dose, administered after completion of the primary series. For most COVID-19 vaccines, this will be a third dose. Booster doses have been offered to different target groups in order of priority. All of the registered COVID-19 vaccines in the National Vaccination Register for this population were from AstraZeneca, Pfizer, and Moderna.

### Definitions of exposure assessments

The assessments of asthma and asthma-associated characteristics were based on questionnaire and clinical data from the 24-year follow-up. Asthma was defined as having a doctor’s diagnosis of asthma (ever) in combination with symptoms of wheeze in the 12 months preceding the follow-up and/or receipt of asthma medication occasionally or regularly in the 12 months preceding the follow-up.^13^ Asthma control was based on the Global Initiative for Asthma definition,^18^ which included: wheeze daily at least 2 times a week, any nighttime awakening, activity limitation in the preceding 4 weeks, and receipt of a symptom reliever at least twice a week in the preceding 12 months. Uncontrolled asthma was defined as meeting at least 1 of 4 criteria. The definition of asthma including >12 episodes of wheeze is provided in this article’s Online Repository at www.jaci-global.org. Further, receipt of inhaled corticosteroids (ICS) was based on self-reported information regarding the 12 months preceding the 24-year follow-up. Fractional exhaled nitric oxide (Feno) of ≥25 ppb indicated the presence of airway inflammation; the method has been described in detail elsewhere.^11^ Values for blood eosinophil cell count were regarded as above reference values if ≥0.3 × 10^9^ cells/L. Definitions of rhinitis and IgE sensitization to common inhalant and food allergens have been given in detail elsewhere^11^ and are provided in the Online Repository.

### Assessment of covariates

Information on early life factors was obtained from the baseline questionnaire (age, sex, parental education, parental socioeconomic status, parent born outside Sweden, maternal age at birth, tobacco smoke exposure, breast-feeding, family history of allergic disease, and siblings). The definitions are provided in the Online Repository, and they have also been given in detail elsewhere.^11^^,^^19^

Lifestyle factors were derived from the 24-year follow-up including occupation (studying, employed, or other, including parental leave or leave of absence, unemployed), educational level (university or elementary/high school),^13^ smoking (daily/occasionally smoking or nonsmoking),^20^ and body mass index (BMI) based on weight and height measured at clinical examination (BMI <25 kg/m^2^, 25-29.9 kg/m^2^, or BMI ≥30 kg/m^2^),^13^ general stress based on the perceived stress scale (PSS-10)^21^ (details provided in the Online Repository), and self-reported amount of physical activity in the preceding 12 months categorized based on the World Health Organization (WHO) recommendations on physical activity.^22^ Details have been provided elsewhere.^23^ General health was defined through the question: “How healthy do you consider yourself to be?” (not very healthy/fairly healthy or completely healthy).^23^

COVID-19–related factors were derived from the COVID-19 phase 3 questionnaire. Confirmed COVID-19 was defined as any of: self-reported SARS-CoV-2 positive PCR test, antigen test, or IgG test (before vaccination) and/or positive PCR test in SmiNet (a national register for notifiable transmittable diseases in Sweden, covering all positive PCR tests).^24^^,^^25^ After COVID-19, it was defined as at least 1 symptom (details in the Online Repository) lasting for at least 2 months after COVID-19 in combination with confirmed COVID-19. Adaption of behavior to reduce the spread of COVID-19, when such recommendations applied, and concerns related to COVID-19 were analyzed through questions included in the Online Repository.

### Statistical analysis

Descriptive data are presented as numbers and percentages for categorical variables (early life, lifestyle, and COVID-19–related factors, asthma, asthma-associated characteristics, and COVID-19 vaccine doses received, and as means and standard deviations for PSS-10 scores. To analyze differences between COVID-19 vaccine doses with regard to early life, lifestyle, and COVID-19–related factors, the Pearson chi-square and Fisher exact tests were used for categorical variables and ANOVA for the PSS-10 score. *P* < .05 was considered statistically significant.

**Fig 2:**
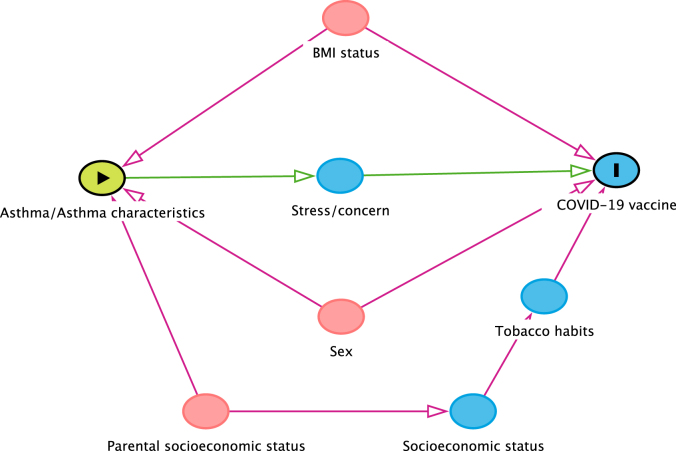
Potential confounders and mediators of relationship between asthma and asthma-associated characteristics at 24-year follow-up, and COVID-19 vaccine uptake, visualized in directed acyclic graph.

The association between number of COVID-19 vaccine doses and asthma and asthma-associated characteristics was analyzed by a logistic regression model. Directed acyclic graph methodology^26^ was used to identify and visualize potential confounders and mediators of the relationship between asthma and asthma-associated characteristics (exposures) and number of COVID-19 vaccine doses (outcome). On the basis of the directed acyclic graph, we adjusted for sex, BMI status, and parental socioeconomic status (blue- and white-collar worker) (Fig 2), expressed as odds ratios (ORs) with 95% confidence intervals (CIs).

All combinations of vaccine types were included in the analyses. For the logistic regression analyses in the subpopulation 24-year follow-up, vaccine doses are defined as either ≥2 or ≥3 doses, with the reference 0 dose. For the analyses regarding ≥2 doses compared to 0 dose, 1 dose (n = 80) is coded as missing, and for ≥3 doses compared to 0 dose, 1 and 2 doses (n = 946) are coded as missing. We also performed sensitivity analyses to investigate if the coding of missing had any effect on the association. In these analyses, the reference was 0 or 1 dose compared to ≥2 doses, and 0, 1, or 2 doses compared to ≥3 doses. All analyses were performed by Stata v14.0 software (StataCorp, College Station, Tex).

## Results

### Description of study population and subpopulations

Table E1 in the Online Repository available at www.jaci-global.org presents a description of the original cohort and subpopulations 24-year follow-up and COVID-19 phase 3 in relation to early life factors. The 2 subpopulations had higher proportions of female subjects and participants with parents with at least university education compared to the original cohort. Moreover, each successive subpopulation had increasingly higher vaccine uptake for ≥3 doses (Table I).

**Table I:** Number of COVID-19 vaccine doses in study population and subpopulations

COVID-19 vaccine dose	Study population(N = 4,064)		Subpopulation 24-year follow-up(n = 3,064)		Subpopulation COVID-19 phase 3(n = 2,049)	
	No.	%	No.	%	No.	%
0 doses	573	14.1	331	10.8	164	8.0
1 dose	117	2.9	80	2.6	37	1.8
2 doses	1,182	29.1	866	28.3	531	25.9
≥3 doses	2,192	53.9	1,785	58.3	1,317	64.3

	**Asthma**		**No asthma**		**Asthma**		**No asthma**	
	**No.**	**%**	**No.**	**%**	**No.**	**%**	**No.**	**%**
0 doses	48	13.9	282	10.4	26	11.2	138	7.6
1 dose	11	3.2	69	2.6	3	1.3	34	1.9
2 doses	83	24.0	778	28.8	50	21.6	479	26.4
≥3 doses	204	58.9	1,577	58.3	153	66.0	1,161	64.1

The mean ages of the participants in the subpopulations 24-year follow-up (n = 3,064) and COVID-19 phase 3 (n = 2,049) were 22.5 years (range, 21.5-25.2 years) and 26.5 years (range, 24.9-27.9 years), respectively, when they answered the questionnaires.

### COVID-19 vaccine uptake in relation to early life and lifestyle factors

In the study population (n = 4,064), 14.1% (n = 573) had no COVID-19 vaccine dose registered in the National Vaccination Register (Table I). The uptake of 2 doses was 29.1%, and that for ≥3 doses was 53.9%. It was a difference in vaccine uptake in relation to sex (*P* < .001), where 24.9% of female subjects and 33.2% of male subjects had an uptake of 2 doses, and 59.4% compared to 48.6% had an uptake of ≥3 doses (Table II).

**Table II:** COVID-19 vaccine dose received in relation to early life factors in study population

Early life factor and COVID-19 vaccine dose	Study population (n = 4,064)				
	No.	%	No.	%	*P* value∗
Sex	Female		Male		
0 doses	261	13.0	312	15.2	<.01
1 dose	56	2.8	61	3.0	
2 doses	501	24.9	681	33.2	
≥3 doses	1,196	59.4	996	48.6	
Parental education	Elementary school/high school		University		
0 doses	327	17.1	245	11.4	<.01
1 dose	74	3.9	43	2.0	
2 doses	624	32.7	554	25.8	
≥3 doses	884	46.3	1,306	60.8	
Parental socioeconomic status	Blue collar		White collar		
0 doses	162	23.5	389	11.8	<.01
1 dose	24	3.5	86	2.6	
2 doses	248	36.0	918	27.8	
≥3 doses	255	37.0	1,911	57.8	
Parent born outside Sweden	Yes		No		
0 doses	153	21.5	262	9.8	<.01
1 dose	24	3.4	79	3.0	
2 doses	213	29.9	764	28.5	
≥3 doses	323	45.3	1,573	58.7	
Mother’s age at birth	<25 years		≥25 years		
0 doses	85	26.7	488	13.0	<.01
1 dose	11	3.5	106	2.8	
2 doses	103	32.4	1,079	28.8	
≥3 doses	119	37.4	2,072	55.3	
Parental smoking at baseline	Yes		No		
0 doses	159	18.7	410	12.8	<.01
1 dose	33	3.9	82	2.6	
2 doses	274	32.3	904	28.3	
≥3 doses	383	45.1	1,797	56.3	

∗ Pearson chi-square tests was used to analyze differences between number of COVID-19 vaccination dose and early life factors.

In the study population (n = 4,064), young adults with parents with higher socioeconomic status (at least university education level and/or white-collar worker) had a higher uptake of ≥3 doses compared to participants whose parents had lower socioeconomic status (elementary school/high school and blue-collar worker) (Table II). Lower uptake of ≥3 doses was seen among participants with a mother aged <25 years at birth, parent born outside Sweden, and parental smoking at baseline (all *P* < .01).

When the influence of lifestyle in young adulthood was examined in the subpopulation 24-year follow-up (n = 3,064), it was a difference in participants’ level of education and vaccine uptake (*P* < .001) (Table III). Among participants with university/college education, 65.7% had an uptake of ≥3 vaccine doses compared to 54.1% among the participants with elementary school/high school education level. Those studying had a higher vaccine uptake of ≥3 doses compared to participants employed or “other.” Young adults smoking daily or occasionally had a lower uptake of ≥3 doses compared to nonsmokers. A lower uptake of ≥3 doses was also seen among young adults considering themselves not very healthy/fairly healthy compared to those considering themselves completely healthy. Participants’ BMI status, physical activity, and perceived stress were not significantly related to vaccine uptake.

**Table III:** COVID-19 vaccine dose received in relation to lifestyle factors in subpopulation 24-year follow-up

Lifestyle factors and COVID-19 vaccine dose	Subpopulation 24-year follow-up (n = 3,064)						
	No.	%	No.	%	No.	%	*P* value∗
Occupation	Studying		Employed		Other		
0 doses	123	7.9	171	13.7	34	14.2	<.01
1 dose	26	1.7	46	3.7	7	2.9	
2 doses	358	22.9	427	34.2	78	32.6	
≥3 doses	1,057	67.6	605	48.4	120	50.2	
BMI status	<25 kg/m^2^		25-29.9 kg/m^2^		≥30 kg/m^2^		
0 doses	8	6.3	147	9.1	58	11.2	.15
1 dose	7	5.5	38	2.3	13	2.5	
2 doses	32	25.2	448	27.6	151	29.2	
≥3 doses	80	63.0	991	61.0	295	57.1	

	**No.**	**%**	**No.**	**%**	
Education	Elementary school/high school		University/college		
0 doses	222	11.4	108	9.9	<.01
1 dose	56	2.9	23	2.1	
2 doses	616	31.6	245	22.4	
≥3 doses	1,055	54.1	719	65.7	
Smoking	Daily or occasionally		Nonsmoking		
0 doses	81	12.8	249	10.3	<.01
1 dose	22	3.5	58	2.4	
2 doses	210	33.2	653	27.0	
≥3 doses	320	50.6	1,461	60.4	
Fulfilled physical activity recommendations	Yes		No		
0 doses	206	9.6	42	12.0	.16
1 dose	51	2.4	7	2.0	
2 doses	589	27.6	110	31.4	
≥3 doses	1,291	60.4	191	54.6	
How healthy do you consider yourself to be?	Completely healthy		Not very healthy/fairly healthy		
0 doses	180	9.9	129	11.8	.03
1 dose	37	2.0	35	3.2	
2 doses	502	27.7	317	29.1	
≥3 doses	1,096	60.4	610	55.9	

	**Mean**				**SD**		***P* value**†
PSS-10							
0 doses	15.0				6.8		.07
1 dose	16.4				9.0		
2 doses	16.1				7.0		
≥3 doses	15.1				6.9		

*SD,* Standard deviation.

∗ Pearson chi-square and Fisher exact tests were used to analyze differences between number of COVID-19 vaccination dose and lifestyle factors.

† ANOVA was used to analyze differences between mean PSS-10 points over number of COVID-19 vaccination dose.

### COVID-19 vaccine uptake in relation to asthma and asthma-associated characteristics

In the subpopulation 24-year follow-up (n = 3,064), the prevalence of asthma was 11.3% (n = 346). In a logistic regression analysis, young adults with asthma had decreased odds of receiving ≥2 doses compared to participants without asthma (adjusted OR = 0.62; 95% CI, 0.42-0.92) (Table IV). Decreased odds were also seen for receiving ≥3 doses in the adjusted model. Further, participants with uncontrolled asthma had decreased odds of receiving ≥2 doses compared to peers with controlled asthma (adjusted OR = 0.32; 95% CI, 0.15-0.68). Decreased odds were also seen for receiving ≥3 doses. Young adults with asthma including >12 episodes of wheeze, asthma, and high eosinophil counts, or asthma and IgE sensitization all had decreased odds of receiving ≥2 and ≥3 doses in the adjusted models compared to peers without asthma and those characteristics.

**Table IV:** COVID-19 vaccine dose received in relation to asthma and asthma-associated characteristics in subpopulation 24-year follow-up (n = 3,064)

Asthma, asthma-associated characteristics, and COVID-19 vaccine dose	No.∗	Crude		Adjusted†	
		OR	95% CI	OR	95% CI
Asthma (reference: no asthma)					
≥2 doses‡	287	0.72	0.52-1.00	0.62	0.42-0.92
≥3 doses‡	204	0.76	0.54-1.07	0.62	0.41-0.92
Uncontrolled asthma (reference: controlled asthma)					
≥2 doses	86	0.50	0.26-0.94	0.32	0.15-0.68
≥3 doses	55	0.43	0.22-0.83	0.30	0.13-0.66
Asthma including >12 episodes of wheeze (reference: no asthma or asthma with ≤12 episodes of wheeze)					
≥2 doses	223	0.71	0.49-1.02	0.55	0.36-0.84
≥3 doses	161	0.76	0.53-1.11	0.56	0.36-0.86
Asthma with ICS regularly for at least 2 months (reference: no asthma with ICS)					
≥2 doses	27	0.41	0.13-1.28	0.27	0.06-1.21
≥3 doses	18	0.36	0.11-1.18	0.21	0.04-1.12
Asthma with Feno ≥ 25 ppb (reference: no asthma or asthma with Feno < 25 ppb)					
≥2 doses	57	0.80	0.36-1.80	0.77	0.34-1.75
≥3 doses	40	0.82	0.36-1.87	0.76	0.33-1.75
Asthma with blood eosinophil cell count ≥0.3 × 10^9^ cells/L (reference: no asthma or asthma with blood eosinophil cell count <0.3 × 10^9^ cells/L)					
≥2 doses	45	0.39	0.20-0.75	0.38	0.20-0.75
≥3 doses	35	0.44	0.22-0.86	0.41	0.20-0.81
Asthma and rhinitis (reference: no asthma and no rhinitis)					
≥2 doses	181	0.72	0.48-1.09	0.65	0.39-1.06
≥3 doses	127	0.76	0.49-1.15	0.60	0.36-1.02
Asthma and IgE sensitization—inhalant and/or food allergens (reference: no asthma and no IgE sensitization)					
≥2 doses	165	0.55	0.35-0.86	0.54	0.34-0.86
≥3 doses	115	0.55	0.30-0.88	0.51	0.32-0.83

∗ Number of cases with asthma and asthma-associated characteristics.

† Adjusted for sex, BMI status, and parental socioeconomic status.

‡ Results obtained by logistic regression. Reference is 0 doses for all analyses.

The sensitivity analyses with a reference of 0 and 1 COVID-19 vaccine doses showed similar results, with decreased odds of receiving ≥2 doses among participants with asthma compared to young adults without asthma in the adjusted model. Moreover, the sensitivity analyses with a reference of 0, 1, and 2 doses also showed decreased odds for receiving ≥3 doses among participants with asthma compared to participants without asthma in the adjusted model, although the difference was not statistically significant (see Table E2 in the Online Repository at www.jaci-global.org).

### COVID-19 vaccine uptake among participants with asthma in relation to COVID-19–related factors

In the subpopulation COVID-19 phase 3 (n = 2,049), the prevalence of asthma was 11.4% (n = 232). When the influence of COVID-19–related factors was examined, a difference was found in vaccine uptake in relation to asthma among those with increased concerns about own health due to COVID-19 (*P* = .006) (Table V). Among young adults with increased concerns about their own health, 13.1% of those with asthma had no COVID-19 vaccine dose registered compared to 7.4% of those without asthma; 17.1% had an uptake of 2 doses compared to 26.5% of those without asthma; and 68.8% had an uptake of ≥3 doses compared to 64.4% of those without asthma. Among those with increased concerns due to COVID-19 or for family or relatives, there was no difference in vaccine uptake in relation to asthma. It was a difference in vaccine uptake in relation to asthma among those with confirmed COVID-19 (*P* = .004). Among the participants with asthma and with confirmed COVID-19, 16.0% had an uptake of 2 doses compared to those 28.4% among the participants without asthma and with confirmed COVID-19.

**Table V:** COVID-19 vaccine dose received in relation to asthma and COVID-19–related factors in subpopulation COVID-19 phase 3 (n = 2,049)

COVID-19–related factors and COVID-19 vaccine dose	Asthma		No asthma		*P* value∗
	No.	%	No.	%	
Confirmed COVID-19: Yes					
0 doses	11	14.7	36	6.0	.004
1 dose	3	4.0	9	1.5	
2 doses	12	16.0	172	28.4	
≥3 doses	49	65.3	388	64.1	
After COVID-19: Yes					
0 doses	2	12.5	6	6.3	.67
1 dose	0	0	1	1.1	
2 doses	4	25.0	25	26.3	
≥3 doses	10	62.5	63	66.3	
Increased concern due to COVID-19: Yes					
0 doses	18	12.0	93	7.5	.20
1 dose	2	1.3	23	1.9	
2 doses	33	22.0	335	27.1	
≥3 doses	97	64.7	785	63.5	
Increased concern for family or close relative’s health due to COVID-19: Yes					
0 doses	10	12.2	59	8.1	.17
1 dose	1	1.2	10	1.4	
2 doses	15	18.3	205	28.0	
≥3 doses	56	68.3	459	62.6	
Increased concern about own health due to COVID-19: Yes					
0 doses	23	13.1	110	7.4	.006
1 dose	2	1.1	26	1.8	
2 doses	30	17.1	393	26.5	
≥3 doses	121	68.8	955	64.4	
Stayed at home with symptoms of COVID-19: Yes, to a large extent					
0 doses	19	11.1	82	6.4	.13
1 dose	3	1.7	18	1.4	
2 doses	36	20.9	305	23.7	
≥3 doses	114	66.3	882	68.5	
Used face mask when unable to keep distance: Yes, to a large extent					
0 doses	9	13.0	50	10.3	.57
1 dose	3	4.4	12	2.5	
2 doses	11	15.9	96	19.7	
≥3 doses	46	66.7	329	67.6	
Avoided crowds, eg, when shopping: Yes, to a large extent					
0 doses	8	8.5	50	6.8	.87
1 dose	2	2.1	16	2.2	
2 doses	20	21.3	172	23.2	
≥3 doses	64	68.1	502	67.8	
Refrained from going to restaurants or visiting shopping centers: Yes, to a large extent					
0 doses	6	6.7	36	5.4	.84
1 dose	1	1.1	11	1.7	
2 doses	17	18.9	150	22.6	
≥3 doses	66	73.3	466	70.3	

∗ Pearson chi-square and Fisher exact tests were used to analyze differences between number of COVID-19 vaccination dose and COVID-19–related factors in relation to asthma.

These COVID-19–related factors are presented in Table E3 in the Online Repository at www.jaci-global.org.

## Discussion

In this study based on data from a population-based prospective cohort with linkage to the mandatory National Vaccination Register, we observed that 14.6% of the participants had no COVID-19 vaccine dose registered. Further, we found that young adults with asthma or with uncontrolled asthma were less likely to receive 2 or 3 doses or more compared to participants without asthma or with controlled asthma, respectively. In general, young adults from households with lower socioeconomic status had lower vaccine uptake than young adults from households with higher socioeconomic status.

The WHO classified the outbreak of COVID-19 as a pandemic in March 2020,^27^^,^^28^ and COVID-19 vaccination has substantially altered the course of the pandemic.^29^ Globally, by May 25, 2023, 70% of the eligible population have received at least 1 dose of a COVID-19 vaccine.^30^ For the age group 18 to 49 years in Sweden, the Public Health Agency of Sweden as of March 1, 2023, no longer recommends booster doses of the vaccine. However, before then, the recommendation was a primary series of 2 doses and 1 booster dose.^31^ In 2022, the WHO recommended use of COVID-19 booster vaccines, especially for at-risk groups, to reduce the transmission and severity of COVID-19 infection while increasing the titer of antibodies against different types of coronavirus variants.^32^ The WHO targeted a coverage of booster vaccines of at least 70% and 100% for elderly, vulnerable people and health workers. Our results suggest that the coverage of COVID-19 first booster vaccination among young adults in Sweden is lower than the target (53.9%).

For many young adults, COVID-19 vaccination was their first vaccination decision regarding a disease of pandemic status. There are important knowledge gaps about vaccination intentions in young adult populations.^33^ In this study, we identified that a lower parental socioeconomic status seemed to be associated with lower COVID-19 vaccine uptake among young adults. A lower level of educational attainment among young adults has been shown to be associated with COVID-19 vaccine uncertainty and refusal in a previous study,^33^ as well as in this study.

Vaccine hesitancy has been identified by the WHO as one of the 10 greatest threats to global health.^34^ One reason for vaccine hesitancy may be that younger adults have lower concerns of hospitalization and mortality than older adults and thus may perceive receiving a COVID-19 vaccine as less personally beneficial.^9^ To increase vaccination intentions and uptake in this age group, highlighting messages of altruism and the protection of others rather than oneself could be effective.^9^ However, increased concern for family or relatives’ health because of COVID-19 did not seem to make any difference in vaccine uptake in the present study.

Early in the pandemic, a large study from England was conducted to examine factors associated with COVID-19–related death.^35^ Their results showed that—among other factors—severe asthma, defined as asthma with recent receipt of oral corticosteroid therapy, was associated with a higher risk of COVID-19–related death. Another study classified patients with underlying moderate to severe asthma as a high-risk group susceptible to severe COVID-19.^36^ Moreover, it has been suggested that there is a greater risk of SARS-CoV-2 infection and severe clinical outcomes of COVID-19 among patients with allergic rhinitis and asthma, especially nonallergic asthma.^37^ Therefore, our hypothesis was that young adults with asthma would be more likely to be vaccinated.

However, our results showed the opposite, with participants with asthma, including uncontrolled disease, having decreased odds of COVID-19 vaccine uptake. Further, young adults with asthma and asthma-associated characteristics such as >12 episodes of wheeze, high eosinophil counts, and IgE sensitization all also had decreased odds of COVID-19 vaccine uptake. Our results highlight the importance of focusing attention on this group of patients, as a recent systematic review and meta-analysis found that adults ≥18 years with severe asthma requiring high-dose ICS or oral corticosteroids had a higher risk of COVID-19 hospitalization compared to adults with mild asthma and/or adults without asthma.^38^ Still, the recent result from our group showing that mild COVID-19 in young adults with asthma did not affect spirometric lung function^12^ may be reassuring for patients and health care providers. However, the pandemic has profoundly affected the lives of the global population. A recent report from the Swedish National Board of Health and Welfare showed that patients with asthma was the group receiving the lowest proportion of the care they would need during the pandemic.^39^ This report could explain the results of a recent study in our group showing that young adults with asthma reported more COVID-19–related concerns about their own health and perceived stress than peers without asthma.^13^

This study indicates how vaccine intentions are unfolding among young adults, and knowledge about vaccine uptake among various priority populations is needed to optimize public health vaccination messaging in the future. For health care professionals in asthma care, it is important to provide guidance to young adults in their vaccination decisions to ensure high vaccination coverage also in this group of patients.

### Strengths and limitations

Important strengths of the present study include the prospective and population-based design of the BAMSE cohort, as well as the large, well-characterized study sample. Another strength is the use of solid and unique data through linkage to a mandatory Swedish health registry, where reporting is mandatory for all health care providers in Sweden under law, with an expected 100% coverage.^40^ Another strength is the high response rate limiting the risk of selection bias, although this cannot be ruled out entirely, as the subpopulations consisted of more female subjects and participants with parents with higher socioeconomic status compared to the original cohort.

Nonetheless, there are some limitations. Our results are based on the Swedish Public Health Agency’s recommendations on vaccination against COVID-19, and recommendations continually change and vary between countries, as do demographics, health care systems, and the prevalence of asthma. This study may not be representative of the larger global population. However, the principles on COVID-19 vaccination have been consistent worldwide and are of international concern; these results can likely be transferred to other, similar countries and populations. Further, the subpopulation COVID-19 phase 3 ranged from mid to late in the pandemic, so attitudes and behaviors around COVID-19 may have been different for participants surveyed in the middle of the pandemic compared to the late pandemic. Moreover, the definition of vaccination outcome for logistic regression analyses in the present study must be discussed, as follows: 1 dose was coded as missing—not only because of the small number of cases but also because those participants may not be comparable to those not starting vaccination (0 doses), and they may also not be comparable to those starting (2 doses), but not receiving, a booster dose (3 doses). We therefore performed sensitivity analyses, with comparable results. Further, because of the small number of participants with asthma, the power to detect differences was limited in some of the analyses.

### Conclusions

COVID-19 vaccine uptake among young adults is lower in individuals from households with lower socioeconomic status and among those with asthma, including uncontrolled asthma.

## Disclosure statement

Supported by grants from the 10.13039/501100004359Swedish Research Council, the 10.13039/501100006636Swedish Research Council for Health, Working Life and Welfare, 10.13039/501100001862Formas, Swedish Asthma and Allergy Research Foundation, the Swedish Heart–Lung Foundation, and Region Stockholm (ALF project, and for cohort and database maintenance).

Disclosure of potential conflict of interest: E. Melén reports personal fees from AstraZeneca, Chiesi, Sanofi, and Novartis outside the submitted work. The rest of the authors declare that they have no relevant conflicts of interest.
