# Supplementary material for: COVID-19 vaccine uptake among young adults: Influence of asthma and sociodemographic factors
Source: J Allergy Clin Immunol Glob. 2024 Feb 20;3(2):100231. doi: 10.1016/j.jacig.2024.100231 (PMC10959661; doi:10.1016/j.jacig.2024.100231)
Supplement: Supplementary Tables [file mmc1.docx]

**Table E1.** Early life factors in the original cohort and sub-populations.

| ***Early life factors*** | **Original cohort**  N = 4,089, 100% | | **Sub-population** **24-year follow-up**  n = 3,064, 75% of original cohort | | | **Sub-population COVID-19 phase 3**  n = 2,049, 69% of invited^a^ | | |
| --- | --- | --- | --- | --- | --- | --- | --- | --- |
|  | n | % | n | % | 95% CI | n | % | 95% CI |
| **Female sex** | 2,024 | 49.5 | 1,619 | 52.8 | 51.1–54.6 | 1,209 | 59.0 | 56.8–61.1 |
|  |  |  |  |  |  |  |  |  |
| **Parental education – university** | 2,161 | 52.9 | 1,698 | 55.5 | 53.5–56.9 | 1,210 | 59.1 | 57.5–61.7 |
|  |  |  |  |  |  |  |  |  |
| **Parent(s) born outside Sweden** | 718 | 21.1 | 561 | 20.7 | 19.2–22.3 | 365 | 19.6 | 17.9–21.5 |
|  |  |  |  |  |  |  |  |  |
| **Mother’s age at birth < 25 years** | 319 | 7.8 | 220 | 7.2 | 6.3–8.2 | 122 | 6.0 | 5.0–7.1 |
|  |  |  |  |  |  |  |  |  |
| **Parental smoking at baseline** | 855 | 21.0 | 620 | 20.4 | 18.9–21.8 | 381 | 18.7 | 17.0–20.5 |
|  |  |  |  |  |  |  |  |  |
| **Exclusively breastfed** ≥ **4 months** | 3,116 | 79.5 | 2,390 | 80.4 | 78.9–81.8 | 1,632 | 81.4 | 79.6–83.1 |
|  |  |  |  |  |  |  |  |  |
| **Family history of allergic disease** | 1,200 | 29.7 | 927 | 30.5 | 28.9–32.2 | 626 | 30.9 | 28.9–33.0 |
|  |  |  |  |  |  |  |  |  |
| **Any older siblings at baseline** | 1,980 | 48.4 | 1,435 | 46.8 | 45.1–48.6 | 942 | 46.0 | 43.8–48.2 |

^a^An invitation was sent to all participants who had answered the questionnaire at the 24-year follow-up and had provided their e-mail address.

**Table E2**. Sensitivity analyses of COVID-19 vaccine dose(s) received in relation to asthma and asthma-associated characteristics in the sub-population 24-year follow-up (n = 3,064).

| ***Asthma, asthma-associated characteristics,***  ***and COVID-19 vaccine dose(s)*** | **Sub-population 24-year follow-up** | | | | |
| --- | --- | --- | --- | --- | --- |
|  | n = 3,064 | | | | |
|  | n^c^ | OR_crude_ | 95% CI | OR^d^_adjusted_ | 95% CI |
| **Asthma (ref: no asthma)** |  |  |  |  |  |
| ≥ 2 doses^a^ | 287 | 0.73 | 0.54–0.98 | 0.62 | 0.44–0.89 |
| ≥ 3 doses^b^ | 204 | 1.03 | 0.82–1.29 | 0.93 | 0.71–1.21 |
| **Uncontrolled asthma (ref: controlled asthma)** |  |  |  |  |  |
| ≥ 2 doses | 86 | 0.57 | 0.31–1.02 | 0.40 | 0.20–0.78 |
| ≥ 3 doses | 55 | 0.56 | 0.35–0.89 | 0.52 | 0.30–0.89 |
| **Asthma including > 12 episodes of wheeze  (ref: no asthma or asthma with ≤ 12 episodes of wheeze)** |  |  |  |  |  |
| ≥ 2 doses | 223 | 0.71 | 0.51–0.99 | 0.57 | 0.38–0.83 |
| ≥ 3 doses | 161 | 1.06 | 0.82–1.37 | 0.91 | 0.68–1.22 |
| **Asthma with ICS regularly for at least 2 months (ref: no asthma with ICS)** |  |  |  |  |  |
| ≥ 2 doses | 27 | 0.44 | 0.15–1.26 | 0.37 | 0.10–1.31 |
| ≥ 3 doses | 18 | 0.55 | 0.25–1.21 | 0.52 | 0.21–1.31 |
| **Asthma with FeNO ≥ 25 ppb (ref: no asthma or asthma with FeNO < 25 ppb)** |  |  |  |  |  |
| ≥ 2 doses | 57 | 0.70 | 0.35–1.40 | 0.67 | 0.33–1.35 |
| ≥ 3 doses | 40 | 0.94 | 0.57–1.55 | 0.97 | 0.58–1.61 |
| **Asthma with blood eosinophil cell count ≥ 0.3 × 10^9^ cells/**L **(ref: no asthma or asthma with blood eosinophil < 0.3 × 10^9^ cells/**L**)** |  |  |  |  |  |
| ≥ 2 doses | 45 | 0.49 | 0.25–0.93 | 0.47 | 0.24–0.92 |
| ≥ 3 doses | 35 | 1.02 | 0.59–1.75 | 1.04 | 0.60–1.80 |
| **Asthma and rhinitis (ref: no asthma and no rhinitis)** |  |  |  |  |  |
| ≥ 2 doses | 181 | 0.77 | 0.52–1.13 | 0.67 | 0.42–1.05 |
| ≥ 3 doses | 127 | 1.01 | 0.76–1.35 | 0.88 | 0.63–1.23 |
| **Asthma and IgE sensitization** – **inhalant and/or food allergens (ref: no asthma and no IgE sensitization)** |  |  |  |  |  |
| ≥ 2 doses | 165 | 0.60 | 0.40–0.91 | 0.59 | 0.38–0.90 |
| ≥ 3 doses | 115 | 0.85 | 0.62–1.15 | 0.85 | 0.62–1.16 |

^a^Results obtained with logistic regression. The reference is 0 and 1 doses compared with ≥ 2 doses. ^b^The reference is 0, 1 and 2 doses compared with ≥ 3 doses. ^c^Number of cases with asthma and asthma-associated characteristics. ^d^Adjusted for sex, BMI status, and parental socioeconomic status.

**Table E3.** COVID-19 vaccine dose(s) received in relation to COVID-19-related factors in the sub-population COVID-19 phase 3 (n = 2,049).

| ***COVID-19-related factors and COVID-19 vaccine dose(s)*** | **Sub-population COVID-19 phase 3** | | | | |
| --- | --- | --- | --- | --- | --- |
|  | n = 2,049 | | | | |
|  | n | % | n | % | p value^a^ |
| **Confirmed COVID-19** | ***Yes*** | | ***No*** | |  |
| 0 dose | 47 | 6.9 | 115 | 8.6 | 0.56 |
| 1 dose | 12 | 1.8 | 25 | 1.9 |  |
| 2 doses | 184 | 26.9 | 341 | 25.4 |  |
| ≥ 3 doses | 440 | 64.4 | 862 | 64.2 |  |
| **Post-COVID-19** | ***Yes*** | | ***No*** | |  |
| 0 dose | 8 | 7.1 | 156 | 8.1 | 0.97 |
| 1 dose | 1 | 0.9 | 36 | 1.9 |  |
| 2 doses | 29 | 25.9 | 501 | 26.0 |  |
| ≥ 3 doses | 74 | 66.1 | 1,236 | 64.1 |  |
| **Increased concern due to COVID-19** | ***Yes*** | | ***No*** | |  |
| 0 dose | 52 | 8.7 | 111 | 8.0 | 0.57 |
| 1 dose | 8 | 1.3 | 25 | 1.8 |  |
| 2 doses | 144 | 24.2 | 369 | 26.6 |  |
| ≥ 3 doses | 392 | 65.8 | 885 | 63.7 |  |
| **Increased concern for family or close relative’s health due to COVID-19** | ***Yes*** | | ***No*** | |  |
| 0 dose | 94 | 8.1 | 69 | 8.4 | 0.59 |
| 1 dose | 22 | 1.9 | 11 | 1.3 |  |
| 2 doses | 292 | 25.0 | 221 | 27.0 |  |
| ≥ 3 doses | 759 | 65.0 | 517 | 63.2 |  |
| **Increased concern about own health due to COVID-19** | ***Yes*** | | ***No*** | |  |
| 0 dose | 30 | 9.3 | 133 | 8.0 | 0.67 |
| 1 dose | 28 | 1.7 | 5 | 1.6 |  |
| 2 doses | 89 | 27.6 | 424 | 25.5 |  |
| ≥ 3 doses | 198 | 61.5 | 1,079 | 64.8 |  |
| **Stayed at home with symptoms of COVID-19** | ***Yes, to a large extent*** | | ***No/yes, to some extent*** | |  |
| 0 dose | 101 | 6.9 | 62 | 11.9 | < 0.01 |
| 1 dose | 21 | 1.4 | 12 | 2.3 |  |
| 2 doses | 343 | 23.4 | 170 | 32.5 |  |
| ≥ 3 doses | 998 | 68.2 | 279 | 53.4 |  |
| **Used face mask when unable to keep distance** | ***Yes, to a large extent*** | | ***No/yes, to some extent*** | |  |
| 0 dose | 59 | 10.6 | 104 | 7.3 | < 0.01 |
| 1 dose | 15 | 2.7 | 18 | 1.3 |  |
| 2 doses | 109 | 19.5 | 402 | 28.2 |  |
| ≥ 3 doses | 376 | 67.3 | 903 | 63.3 |  |
| **Avoided crowds, e.g., when shopping** | ***Yes, to a large extent*** | | ***No/yes, to some extent*** | |  |
| 0 dose | 58 | 6.9 | 105 | 9.2 | 0.008 |
| 1 dose | 18 | 2.2 | 15 | 1.3 |  |
| 2 doses | 193 | 23.1 | 318 | 27.7 |  |
| ≥ 3 doses | 568 | 67.9 | 709 | 61.8 |  |
| **Refrained from going to restaurants or visiting shopping centers** | ***Yes, to a large extent*** | | ***No/yes, to some extent*** | |  |
| 0 dose | 42 | 5.6 | 121 | 9.9 | < 0.01 |
| 1 dose | 12 | 1.6 | 21 | 1.7 |  |
| 2 doses | 169 | 22.4 | 341 | 27.8 |  |
| ≥ 3 doses | 533 | 70.5 | 746 | 60.7 |  |

^a^Pearson’s chi-squared and Fisher exact tests were used to analyze differences between number of COVID-19 vaccination dose(s) and COVID-19-related factors.
