# Supplementary material for: COVID-19 vaccine uptake among young adults: Influence of asthma and sociodemographic factors
Source: J Allergy Clin Immunol Glob. 2024 Feb 20;3(2):100231. doi: 10.1016/j.jacig.2024.100231 (PMC10959661; doi:10.1016/j.jacig.2024.100231)
Supplement: Supplementary Material [file mmc2.docx]

**Online Repository**

**COVID-19 vaccine uptake among young adults – influence of asthma and sociodemographic factors**

Maria Ödling, Ph.D.^1^, Niklas Andersson, MSc.^2^, Sandra Ekström, Ph.D.^1, 2, 3^, Niclas Roxhed, Ph.D.^4, 5^, Jochen M. Schwenk, Ph.D.^6^, Sophia Björkander, Ph.D.^1^, Anna Bergström, Ph.D.^2, 3^, Erik Melén, M.D., Ph.D.^1, 7^, Inger Kull, Ph.D.^1, 7^ & on behalf of the BAMSE COVID-19 study group^

1. Department of Clinical Science and Education, Södersjukhuset, Karolinska Institutet,

Stockholm, Sweden;

1. Institute of Environmental Medicine, Karolinska Institutet, Stockholm, Sweden;
2. Centre for Occupational and Environmental Medicine, Region Stockholm, Stockholm, Sweden;
3. Division of Micro and Nanosystems, KTH Royal Institute of Technology, Stockholm, Sweden;
4. MedTechLabs, Bioclinicum, Karolinska University Hospital, Solna, Sweden;
5. Science for Life Laboratory, Department of Protein Science, KTH Royal Institute Technology, Solna, Sweden;
6. Sachs’ Children and Youth Hospital, Stockholm, Sweden.

^Membership in the BAMSE COVID-19 study group is stated in the Acknowledgements.

**Corresponding author**

Maria Ödling

Forskningscentrum

Södersjukhuset

SE-118 83 Stockholm, Sweden

Telephone: +468 524 800 02

E-mail: Maria.Odling@ki.se

**Methods**

**Asthma characteristics**

The definition of “*asthma including >12 episodes of wheeze*” was based on questionnaire data from the 24-year follow-up and defined as having a doctor’s diagnosis of asthma (ever) in combination with >12 episodes of wheeze in the 12 months preceding the follow-up and/or use of asthma medication occasionally or regularly in the 12 months preceding the follow-up. The assessments of *rhinitis* and *IgE sensitization* were based on questionnaire and clinical data from the 24-year follow-up. Rhinitis was defined as participant’s report of symptoms of sneezing, a runny or blocked nose, and/or itchy, red, and watery eyes after exposure to furred animals or pollen (in the preceding 12 months) and/or doctor’s diagnosis of allergic rhinitis ever up to the date of the 24-year questionnaire. IgE sensitization to common inhalant and food allergens was analyzed using the ImmunoCAP System (Thermo Fisher/Phadia AB, Uppsala, Sweden) with Phadiatop^®^ (cat, dog, horse, birch, timothy, mugwort, *Dermatophagoides pteronyssinus*, and *Cladosporium herbarum*), and fx5^®^ (cow’s milk, peanut, hen’s egg, wheat, soybean, and fish). IgE values greater than 0.35 kU_A_/L were regarded as positive.

**Early life factors**

Early life factors were gathered from the baseline questionnaire answered by the parents when the participant were about 2 months of age. *Parental education* was based on the highest education level of the household. *Parental socioeconomic status* was defined as the dominant socioeconomic status for the household, dichotomized into blue (low) and white (high) collar worker. Based on the Nordic standard occupational classification and Swedish socio-economic classification (1). *Parent born outside Sweden*, was defined as father and/or mother born outside of Sweden. *Tobacco smoke exposure* was defined as either of the parents smoked at least one cigarette per day. *Family history of allergic disease* was defined as mother and/or father with doctor’s diagnosis of asthma and asthma medication and/or doctor’s diagnosis of rhinitis in combination with reported allergy to furred pets and/or pollen.

**Lifestyle factors**

Using questionnaire data from the 24-year follow-up, *general stress* was defined on the basis of the perceived stress scale (PSS-10) consisting of 10 questions regarding how the participants have perceived and handled stress and stressful situations in the preceding month (2). Each question had five response options from “never” to “very often,” which corresponded to 0–4 points (scores were reversed for four questions with positive statements). The total score was 0–40 points.

**COVID-19-related factors**

*COVID-19-related factors* were derived from the COVID-19 phase 3 questionnaire. Participants with confirmed or suspected COVID-19 were asked about long-term symptoms after the COVID-19 infection “Have you had long-term symptoms after the COVID-19 infection (post-COVID/long COVID)?” Participants who answered “Yes” to this question were asked which long-term symptoms they had had and whether the duration of each symptom was ≥2 months or ≥3 months. The long-term symptoms included were: dyspnea (breathing difficulties or shortness of breath), fatigue (extreme physical and/or mental tiredness), fever (or feeling feverish), altered sense of smell or taste, headache, tachycardia (high resting heart rate or palpitations), cognitive impairment (e.g., memory and concentration difficulties), gastrointestinal problems, muscle weakness, neurological symptoms (e.g., numbness), psychiatric symptoms (e.g., depression, anxiety, or feeling down), pain (e.g., chest pain or muscle and joint pain), and sleep disorders. *Post-COVID-19* was defined as at least one symptom lasting for at least 2 months after COVID-19 in combination with confirmed COVID-19.

*Concern* related to COVID-19 was analyzed through the following questions: “Have you felt increased concern due to COVID-19?”, “Have you felt increased concern about the health of your family/close relatives due to COVID-19?”, “Have you felt increased concern about your own health due to COVID-19?”. The response options were yes and no (3). *Adaption of behavior* to reduce the spread of COVID-19 when such recommendations applied was analyzed through the following questions: “Have you stayed at home with symptoms of COVID-19?”, “Have you used a face mask when unable to keep distance?”, “Have you avoided crowds, e.g., when shopping?”, “Have you refrained from going to restaurants or visiting shopping centers?”. The response options were categorized as: No/Yes, To some extent/Not relevant to me, and Yes, to a large extent.

**References**

1. Occupations in Population and Housing Census 1985 (foB 85) according to Nordic standard occupational classification (Nordisk yrkesklassificering, NYK) and Swedish socio-economic classification (Socioekonomisk indelning, SEI) (Swedish). Stockhom. Sweden, 1989.

2. Ekström S, Andersson N, Lövquist A, Lauber A, Georgelis A, Kull I, et al. COVID-19 among young adults in Sweden: self-reported long-term symptoms and associated factors. Scandinavian journal of public health. 2021:14034948211025425.

3. Ekström S, Mogensen I, Georgelis A, Westman M, Almqvist C, Melén E, et al. General Stress Among Young Adults with Asthma During the COVID-19 Pandemic. The journal of allergy and clinical immunology In practice. 2022;10(1):108-15.
